# Synthetic Plasma Liquid Based Electronic Circuits Realization-A Novel Concept

**Published:** 2016-09

**Authors:** Killol V. Pandya, ShivPrasad Kosta

**Affiliations:** Department of electronics and communication, CHARUSAT University, Gujarat, India

**Keywords:** Synthetic plasma, biomedical science, human body

## Abstract

Biomedical research is contributing significant role in the field of biomedical engineering and applied science. It brings research and innovations to a different level. This study investigated artificial human blood –synthetic plasma liquid as conductive medium. Keeping in mind the conductivity of synthetic plasma, astable multivibrator as well as differential amplifier circuit were demonstrated. The circuits were given normal input voltages at regular temperature and ideal conditions. The result shows desired response which supports the novel concept. For both the circuits, phase shift of 180° achieved by analysing biological electronic circuits.

## INTRODUCTION

Majority of human body consists of water and blood particles like white cell, red cell, platelets. Liquid blood is responsible for supply of nutrients such as glucose, amino acids, and fatty acids (dissolved in the blood or bound to plasma proteins (e.g., blood lipids)). An artificial human blood named synthetic plasma was made in the laboratories of applied science to investigate and analyse its behaviour in order to realise liquid based electronic circuits.

Human blood based liquid memristor is made possible and feasible for research and analysis (1). Electronic diode, electronic field effect transistor and gate circuitry is possible to design with the help of human tissue (3). Two human palm fingers were used for the investigation and three rings were designed to acts as gate, sourse and drain terminals. Keeping ring diameter, size and ideal pressure, field effect transistor characteristics were successfully achieved (4).

Physical model of human tissue skin based memoristor and their Network is possible for biomedical analysis (2). This literature supports novel idea to design electronic circuit from synthetic plasma liquid. There is a relation between total body capacitance (TBC) and basal metabolic rate (BMR) because of the electrical properties of human body parts (6). So it is very clear that human body parts can be treated as different electronic components.

The human body can be analyzed as a transmission medium for electrical current by means of numerical simulations and measurements. Properties of predefined tissue layer and variations related to human body geometry were analysed. Significant transmission achieved between two thorax with typical signal to noise ratio (7). Human body can be used as most desiring short range transmission medium for wireless body area network. A novel S-TDMA protocol had been proposed which meets the delay and transmission requirements for human body communication (8).

## MATERIALS AND METHODS

### Biological resistance R and capacitance C

Synthetic plasma has electrically charged particle ions. Under the effect of external emf, they acquire dynamism, face collisions and thus manifest resistance R. Similar manner, electrically charged particle ions are distantly placed have dynamic emf field between them and thus manifest capacitance C. Silicon rubber tubes of various distances with different diameter were filled with synthetic plasma. Tube capacitance C and resistance R were realized by capacitor meter and multimeter.

**Figure 1:**
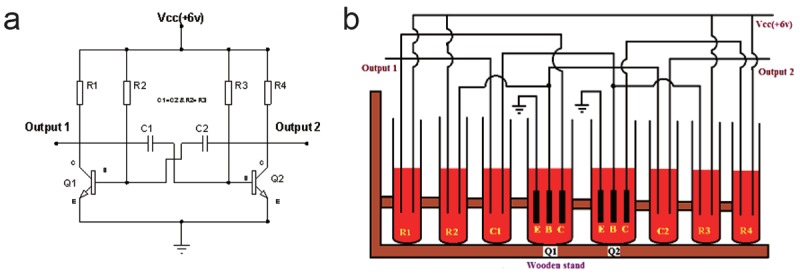
a) Basic astable multivibrator electronc circuit; b) Synthetic plasma circuit layout of astable multivibrator.

Figure 1a depicts basic circuit of astable multivibrator. Here both the capacitors(C_1_ and C_2_ ) are charging and discharging but not at the same time so according their polarities, transistors Q_1_ and Q_2_ are on and off but again not at the same time so as a result, circuit gives two stable states with regular interval.

Designing of human tissue (skin) based transistor is feasible now a days where different point of skin can be treated as three terminals of the transistor (5). Actually, all human body tissues should be naturally rechargeable because there is no possibility to charge them like a battery in actual hardware circuit (9). Also the circuit which deals with human body should be minimum power consumption, compact and less noisy for minimum cell damage (10). The authors have first replaced one register with synthetic plasma and check the output characteristics. Once the output characteristics met with all ideal characteristics of the circuit, authors have replaced another component of the same circuit and eventually they have come up with synthetic plasma liquid based biological electronic circuit.

Figure 1b depicts actual synthetic plasma liquid based basic astable multivibrator circuit. Here, different resistors and capacitors were realised by inserting two metallic wires in to a test tubes (filled with synthetic plasma).Plasma is acting as resistor as well as capacitor as described earlier. After realising resistor and capacitor, successful attempt has been made to realise two transistors. As shown in Figure 1b, three copper plates (2 cm × 3 mm) were shouldered with metallic wires, inserted in test tubes which are filled with plasma, form transistors.

As observed from the figure, all tubes are filled with equal plasma level. For realising registers/capacitors, 15 ml to 20 ml liquid in 12 mm diameter transparent glass test tubes were analysed. Similar manner, realising transistors, 40 ml to 50 ml liquid in 25 mm diameter transparent glass test tubes were analysed. Wooden stand is used for mechanical support.

**Figure 2:**
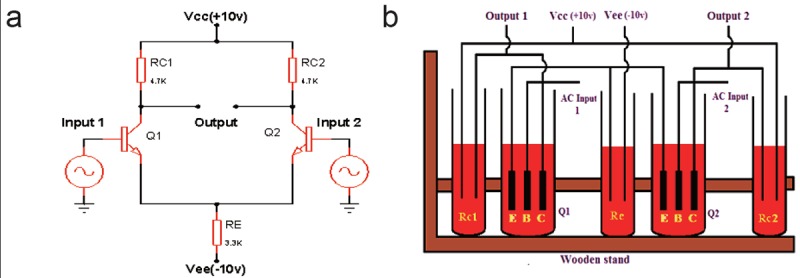
a) Differential amplifier circuit; b) Synthetic plasma circuit layout out of differential amplifier.

Figure 2a and Figure 2b shows electronic differential amplifier circuit and physical model of the similar circuit respectively. In ideal electronic circuit, two bases of transistors are inputs. Hence they subtracted and multiplied by the pair. As a result of this, sinusoidal outputs with 180° phase shift can be obtained.

This time, similar approach has been applied by the authors to achieve electronic circuit. Five tubes were taken to realise three resistors and two transistors as they were filled with synthetic plasma liquid (equal physical quantity). Here also synthetic plasma plays a major role for designing the components and finally whole biological electronic circuit.

## RESULTS AND DISCUSSION

**Figure 3:**
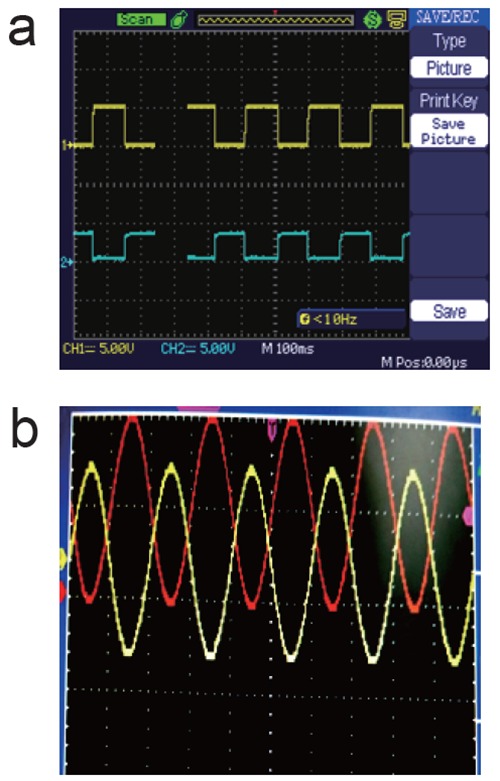
a) Output of astable multivibrator; b) Output of differential amplifier.

Figure 3a and Figure 3b depicts response of proposed biological circuit of astable multivibrator and differential amplifier respectively. Here in Figure 3a, response shows two stable states of two outputs with phase shift of 180°. As described earlier, capacitors play an important role to switch OFF and ON two transistors. In Figure 3b, as AC sinusoidal voltages are given to the base terminals of both the transistors (out of phase) and similar sinusoidal waveforms can be obtained as an output.

The proposed circuits of astable multivibrator and differential amplifier shows desired response which supports feasibility of liquid based electronic circuits. The presented idea can be extended to develop pure biological human implantable circuits which can regulate our body function and heart bits. Of course, research related to human implanting needs critical care to develop similar circuit.

## CONCLUSION

Synthetic plasma liquid has been used as conducting medium to investigate biological electronic circuits (astable multivibrator and differential amplifier). The result supports presented biomedical approach to design biological electronic circuit. Presented circuits are environmentally friendly and support a very novel idea to design pure biological circuit from human blood. Using this novel concept, an extra ordinary research can be made possible to design first human implantable biological electronic circuit. Presented research has very high applications in the field of biomedical science and medical society.
